# Virulome–resistome convergence in swine-associated multidrug-resistant *Escherichia coli* from Hungary: virulence marker profiles and zoonotic potential

**DOI:** 10.3389/fvets.2026.1813532

**Published:** 2026-04-16

**Authors:** Ádám Kerek, Balázs Nagyházi, Gergely Álmos Tornyos, Levente Hunor Husz, Máté Hetyésy, Eszter Kaszab, Enikő Fehér, Patrik Mag, Ákos Jerzsele

**Affiliations:** 1 Department of Pharmacology and Toxicology, University of Veterinary Medicine, Budapest, Hungary; 2 National Laboratory of Infectious Animal Diseases, Antimicrobial Resistance, Veterinary Public Health and Food Chain Safety, University of Veterinary Medicine, Budapest, Hungary; 3 One Health Institute, University of Debrecen, Debrecen, Hungary; 4 Department of Microbiology and Infectious Diseases, University of Veterinary Medicine, Budapest, Hungary; 5 Department of Bioinformatics, University of Debrecen, Debrecen, Hungary

**Keywords:** antimicrobial resistance, *Escherichia coli*, One Health, swine, virulome, whole-genome sequencing

## Abstract

**Background:**

Swine production can sustain dense reservoirs of multidrug-resistant (MDR) *Escherichia coli* in which mobilizable resistance may co-occur with virulence modules relevant to animal health and One Health risk. We profiled the virulome of Hungarian swine-associated MDR *E. coli* and assessed whether key virulence signatures co-segregate with selected high-impact resistance flags.

**Methods:**

In late 2023, *E. coli* isolates were obtained from four large-scale Hungarian pig farms through routine veterinary diagnostic/surveillance sampling. Phenotypic antimicrobial susceptibility testing was performed for the full collection (*n* = 203), including extended-spectrum *β*-lactamase (ESBL) confirmation (*n* = 127). A whole-genome sequencing (WGS) subset (*n* = 116) underwent Illumina sequencing and *de novo* assembly. Virulence genes were identified in silico using curated virulence databases with harmonized identity/coverage thresholds and summarized as gene prevalence and functional modules. Marker-based definitions were applied for extraintestinal pathogenic *E. coli* (ExPEC)-like and diarrheagenic *E. coli* (DEC)-like signatures. Acquired antimicrobial resistance genes were annotated using CARD/RGI, and focused analyses considered *CTX-M* extended-spectrum beta-lactamase (ESBL) genes and rare, high-consequence determinants (*mcr-1*, *qnrB5*). Associations between virulence markers and resistance flags were tested using Fisher’s exact test. Rather than performing a comprehensive resistome analysis, we focused on selected high-impact resistance determinants (*CTX-M*, *mcr-1*, *qnrB5*) and their co-carriage with virulence markers.

**Results:**

Across 116 genomes, 208 distinct virulence-associated genes were detected; virulence gene load per genome was heterogeneous. Extraintestinal-associated iron acquisition modules were common, including aerobactin (*iucABCD*/*iutA*) in 31/116 (26.7%), yersiniabactin (*fyuA/irp1/irp2*) in 27/116 (23.3%), and salmochelin (*iroB/iroN*) in 17/116 (14.7%). Toxin-associated determinants were frequent, with *hlyA* in 33/116 (28.4%). DEC markers occurred in 33/116 (28.4%), including *eae* in 23/116 (19.8%), *stx2* in 6/116 (5.2%), predominantly enterohemorrhagic *E. coli* (EHEC)-like (*stx2* and *eae*) profiles (5/116; 4.3%) and *estIa* in 10/116 (8.6%); *astA* was present in 27/116 (23.3%). Stringent ExPEC-like criteria were met by 6/116 (5.2%), while 15/116 (12.9%) showed convergent profiles co-carrying aerobactin with at least one DEC marker. *CTX-M* genes were detected in 24/116 (20.7%) and were enriched among aerobactin-positive isolates and DEC-marker-positive isolates; all EHEC-like isolates carried *CTX-M*. Rare but critical determinants included *mcr-1* (3/116; 2.6%) and *qnrB5* (2/116; 1.7%).

**Conclusion:**

Hungarian swine-associated MDR *E. coli* show virulome heterogeneity with frequent aerobactin/toxin modules and DEC markers. *CTX-M* enrichment in these profiles indicates virulome–resistome convergence, supporting integrated One Health surveillance.

## 1 Introduction

Antimicrobial resistance (AMR) has become a defining One Health challenge, undermining the effectiveness of antibacterial therapy and increasing the clinical and economic burden of infectious diseases (1, 2). The scale of antimicrobial exposure is not confined to human medicine; the intensification of food-animal production has historically been accompanied by expanded antibiotic use, creating sustained selection pressure in animal-associated bacterial communities (1, 3, 4). Within livestock systems, antimicrobial administration and the high-density ecology of modern production facilitate the emergence and persistence of resistant bacteria and resistance determinants that can ultimately interface with human populations through direct contact, food, and the environment (5–7).

A central driver of AMR dissemination is horizontal gene transfer, which enables bacteria to acquire not only single resistance determinants but also clusters of genes carried by plasmids and other mobile genetic elements (8–11). This gene flow supports the “farm-to-fork” circulation of antimicrobial resistance genes (ARGs) and maintains them within interconnected ecological reservoirs, including animals, manure, soil, water, and food-processing environments (12–15). These interdependencies are explicitly captured by the One Health framework and by international guidance that seeks to preserve medically important antimicrobials while prioritizing effective stewardship across sectors (16, 17).

*Escherichia coli* occupies a pivotal position in this context. It is a dominant member of the intestinal microbiota of humans and animals, serves as an indicator organism in AMR monitoring, and includes pathotypes capable of causing disease across multiple hosts and anatomical niches. The pathogenic spectrum of *E. coli* spans intestinal disease and extraintestinal infections; accordingly, pathotype attribution requires virulence-informed interpretation rather than reliance on species-level identification alone (18–20). In production animals, *E. coli* lineages may circulate as commensals while simultaneously acting as reservoirs of genetic elements relevant to both veterinary and public health, especially when coupled to multidrug resistance (MDR) (21).

From a One Health perspective, the convergence of Extraintestinal pathogenic *E. coli* (ExPEC)- and diarrheagenic *E. coli* (DEC)-associated virulence modules within MDR *E. coli* lineages is epidemiologically relevant because it may broaden the spectrum of host–interaction potential and exposure relevance across animal–environment–food interfaces. ExPEC represents a clinically important group with the capacity to cause disease outside the intestinal tract, including urinary tract infections, septicemia, and systemic syndromes (18, 22, 23). A key conceptual advance is the inclusive ExPEC definition proposed by Russo and Johnson (18), which emphasizes virulence potential as a continuum rather than a narrow syndrome-restricted categorization. In parallel, DEC often discussed under intestinal pathogenic *E. coli* (InPEC), encompasses multiple enteric pathotypes (e.g., Enterotoxigenic *E. coli*, Enteropathogenic *E. coli*, Shiga toxin-producing *E. coli*, Enteroaggregative *E. coli*) that are defined by characteristic virulence gene repertoires and toxin/adhesion marker combinations (20). From a One Health perspective, these categories matter because virulence determinants are frequently mobile or modular, meaning that virulence signatures can disseminate across diverse genomic backgrounds and ecological compartments (8–11).

Phylogenetic background provides additional interpretive power for virulence risk profiling. The Clermont phylogrouping scheme is widely used to stratify *E. coli* into major lineages (e.g., A, B1, B2, D), which differ in their ecological associations and, in many settings, in their propensity to carry particular virulence traits (24). While phylogroup is not a substitute for functional virulence characterization, it offers a tractable population-genetic framework within which virulence marker carriage and resistance determinants can be contextualized, compared, and linked to clonal expansion and transmission dynamics (19, 24). Phylogroup information offers a population-genetic backbone for interpreting whether virulence-marker patterns cluster in lineages that are repeatedly implicated in extraintestinal disease and inter-host transmission, thereby supporting risk-oriented interpretation in livestock-associated *E. coli* populations.

In intensive pig production, these issues intersect with strong antimicrobial selection pressure and with the high epidemiological throughput of large herds. Extended-spectrum *β*-lactamase (ESBL)-producing *E. coli* has become a focal AMR phenotype because ESBL determinants can compromise critically important β-lactams and are frequently mobilized on plasmids that also carry additional resistance genes (25–28). The capacity of *E. coli* to accumulate multi-class resistance determinants is further supported by intrinsic and acquired mechanisms, including efflux systems and other adaptive responses that collectively contribute to MDR phenotypes and persistence under antimicrobial exposure (29–31). Importantly, plasmid-mediated ARG assemblages create a mechanistic substrate for co-selection, whereby selection for one resistance trait can indirectly maintain other linked determinants within the same mobile genetic context (8, 11, 32).

Whole-genome sequencing (WGS) has transformed the surveillance and mechanistic investigation of AMR by enabling simultaneous assessment of phylogenetic background, resistome composition, and (when appropriately curated) virulence gene repertoires at strain-level resolution (33, 34). This integrated view helps bridge phenotypic susceptibility testing with genomic inference, supports higher-resolution epidemiology, and strengthens risk-relevant interpretation beyond single-gene reporting, particularly in reservoirs where resistant strains can disseminate along the food chain and across host boundaries (34–37). For pig-associated *E. coli*, an explicitly convergent analysis of virulence and resistance is therefore critical: the public-health relevance of MDR/ESBL *E. coli* is not determined solely by ARG content, but by how resistance is embedded within specific phylogenetic and virulence backgrounds that may elevate zoonotic risk.

Despite the growing number of WGS-based reports describing MDR/ESBL *E. coli* in intensive pig production, much of the available literature remains resistome-centric, whereas virulence gene content and pathotype-marker distributions are often reported only partially or without systematic functional grouping and phylogenetic context. Moreover, region-specific data from Central Europe, including Hungary, on the distribution of ExPEC- and DEC-associated signatures within swine-associated MDR *E. coli* reservoirs are limited. This gap constrains risk-oriented One Health interpretation, because resistance determinants alone do not capture the virulence architectures that may shape host interaction potential and exposure relevance along animal–environment–food interfaces (25, 26).

Here, we investigate virulome–resistome convergence in a WGS-characterized cohort of swine-associated multidrug-resistant (MDR) *E. coli* from Hungary (*n* = 116 genomes). Our primary aim was to characterize virulence-marker architectures in this production reservoir and evaluate whether key virulence signatures co-segregate with selected high-impact resistance determinants. Specifically, we aimed to quantify virulence gene burden and summarize prevalent virulence determinants by functional module, interpret ExPEC- and DEC-associated marker signatures within phylogroup, MLST backgrounds, and assess co-carriage with selected high-impact resistance flags (*CTX-M* ESBL genes, with targeted consideration of *mcr-1* and *qnrB5*), rather than performing a comprehensive resistome analysis. By anchoring virulence interpretation in phylogenetic context, we provide a risk-oriented genomic baseline for Hungarian intensive pig production to inform future One Health surveillance.

## 2 Materials and methods

### 2.1 Study design, sampling, and bacterial isolates

This retrospective, observational cross-sectional study analyzed archived, culture-positive swine *E. coli* isolates generated during routine veterinary surveillance activities on four large-scale pig farms in Hungary in late 2023 (Nyugat-Dunántúl, Dél-Dunántúl, Dél-Alföld). Submissions reflected surveillance activities and therefore included non-clinical (screening) sampling; systematic animal-level clinical metadata were not uniformly available. When multiple *E. coli* isolates were recovered from the same sample/submission, a single representative isolate was retained for downstream analyses, and repeated isolates were removed where metadata permitted. The researchers received the resulting Amies-type charcoal transport swabs and the corresponding bacterial isolates for downstream investigations. Isolates were transported to the Department of Epidemiology and Microbiology, University of Veterinary Medicine Budapest, where pure cultures were established and forwarded for further analysis. Strains were stored at −80 °C using a bead-based cryopreservation system (Microbank; Pro-Lab Diagnostics, Richmond Hill, Canada).

Phenotypic antimicrobial susceptibility testing was performed on the full cohort (*n* = 203), including ESBL confirmation (*n* = 127 ESBL-positive). A subset of isolates with adequate DNA yield and quality underwent whole-genome sequencing (WGS) and constituted the primary dataset for virulome–resistome analyses (*n* = 116).

The study workflow and isolate selection are summarized in Supplementary Figure S1.

### 2.2 Phenotypic antimicrobial susceptibility testing (MIC)

Minimum inhibitory concentrations (MICs) were determined by broth microdilution following Clinical and Laboratory Standards Institute (CLSI) recommendations, and interpretive breakpoints were applied according to CLSI standards (38). Briefly, isolates stored at −80 °C were revived in cation-adjusted Mueller–Hinton broth (CAMHB) and incubated at 37 °C for 18–24 h. MIC testing was performed in 96-well microtiter plates (VWR International Kft., Debrecen, Hungary) using two-fold serial dilutions of antimicrobial agents prepared as CLSI-compliant stock solutions (1024 μg/mL) and diluted in CAMHB (38). Inocula were adjusted to 0.5 McFarland using a nephelometer and added to wells to achieve standard inoculum density, followed by incubation at 37 °C for 18–24 h. MICs were read using a Sensititre SWIN automated reader and the VIZION system (v3.4; Thermo Fisher Scientific, Budapest, Hungary). Quality control was ensured using *E. coli* ATCC 25922.

### 2.3 Eligibility criteria, deduplication and sample size rationale

We included culture-positive *E. coli* isolates obtained from swine submissions collected during routine veterinary surveillance activities from the participating farms in late 2023. Culture-negative submissions were not part of the study, as the analytical unit was the bacterial isolate. Non-*E. coli* isolates and mixed/impure cultures were excluded. To minimize duplication, when multiple *E. coli* colonies were recovered from the same sample or submission, a single representative isolate was retained for downstream analyses; where available metadata allowed, repeated isolates from the same submission were removed.

No *a priori* sample size calculation was performed because this was an observational study based on a defined, previously established isolate collection. Instead, all eligible isolates available from the study period were included for phenotypic testing (*n* = 203). Whole-genome sequencing was performed on a subset selected on the basis of DNA quality and assembly quality control for downstream virulome analyses (*n* = 116).

### 2.4 Ethical considerations

No animal experiments, interventions, or additional sampling were performed for this study. The work was conducted exclusively on archived bacterial isolates and de-identified metadata generated through routine surveillance activities; therefore, ethical approval and an approval number were not applicable.

### 2.5 Phenotypic ESBL screening and confirmation

ESBL production was assessed using a CLSI-recommended approach (38). MICs were determined for cefotaxime alone and for cefotaxime plus clavulanic acid, maintaining a constant clavulanate concentration of 4 μg/mL across dilutions. Isolates were classified as ESBL-positive when the cefotaxime MIC in the presence of clavulanate decreased by ≥3 two-fold dilutions (i.e., an ≥8-fold reduction) compared with cefotaxime alone, consistent with CLSI guidance (38). Potential false-positive phenotypes due to *β*-lactamase hyperproduction driven by non-ESBL determinants (e.g., *ampC/ampH* overexpression) were considered during interpretation.

### 2.6 Genomic DNA extraction, library preparation, and sequencing

Genomic DNA was extracted using the Zymo Quick-DNA Fungal/Bacterial Miniprep Kit (Zymo Research, Irvine, CA, USA) according to the manufacturer’s protocol (39). Mechanical lysis was performed by bead beating using a TissueLyzer LT (Qiagen, Hilden, Germany) at 50 Hz for 5 min. Extracted DNA was stored at −20 °C until library preparation.

Paired-end sequencing was performed by Novogene on an Illumina NextSeq 500 platform. The sequencing-by-synthesis principle and general Next-Generation Sequencing workflow were considered as described previously (40). Libraries were prepared using the Nextera XT DNA Library Preparation Kit with Nextera XT Index Kit v2 Set A (Illumina, San Diego, CA, USA) (41). Input DNA was normalized to 0.2 ng/μL (2.5 μL total), followed by tagmentation (55 °C, 6 min), neutralization, and index PCR amplification (12 cycles; 95 °C denaturation, 55 °C annealing, 72 °C extension). Indexed libraries were purified using the Geneaid Gel/PCR DNA Fragments Extraction Kit (Geneaid Biotech, New Taipei City, Taiwan) and quantified with the Qubit dsDNA HS Assay (Thermo Fisher Scientific, Waltham, MA, USA). Individually indexed libraries were pooled at equimolar ratios prior to sequencing.

### 2.7 Bioinformatic processing, assembly, and genome quality control

Raw read quality was assessed using FastQC v0.11.9 (42), fastp v0.23.2–3 (43), and Bloocoo v1.0.7 (44). Adapter and low-quality sequence removal was performed with Trim Galore v0.6.6 (45). High-quality reads were assembled *de novo* using both MEGAHIT v1.2.9 (46) and SPAdes v4.0.0 (47), and assemblies were merged using GAM-NGS v1.1b to obtain a robust draft genome per isolate (48). This merged-assembly strategy was used to increase robustness of draft assemblies across heterogeneous isolates and to support reliable recovery of accessory loci relevant to virulence/resistance screening; all assemblies were subsequently evaluated with standard quality metrics prior to inclusion in downstream analyses. Assembly quality and completeness were evaluated with QUAST v5.2 (49) and BUSCO v5 (50). Genome characteristics (e.g., genome size and coverage proxies) were estimated using GenomeScope v2.2 based on k-mer distributions (51). Open reading frames were predicted using Prodigal v2.6.3 (52). Species assignment and contamination screening were performed using CheckM v1.2.2 (53) and Kraken v1.1.1 (54).

### 2.8 Resistome annotation, mobile genetic element and plasmid inference

Acquired antimicrobial resistance genes (ARGs) were identified from assemblies using Resistance Gene Identifier (RGI) v5.1.0 against the Comprehensive Antibiotic Resistance Database (CARD) (55). Only high-confidence ARG calls meeting stringent criteria were retained (CARD “STRICT” category; ≥90% sequence identity and ≥90% coverage).

To assess mobility context, ARG proximity to mobile genetic elements (MGEs) was evaluated using MobileElementFinder v1.0.3 (56). An ARG was considered MGE-associated if located within the maximum transposon-distance threshold defined by the underlying MobileElementFinder database for the organism. Putative plasmid origin of contigs was inferred using PlasFlow v1.1 (57). For both MGE and plasmid assignments, only predictions supported by ≥10 kb of classified sequence per contig were retained to reduce false positives.

### 2.9 Virulome profiling and pathotype assignment

Virulence genes were screened from assemblies using ABRicate (version 1.2.0) with the VFDB database, applying thresholds of ≥90% nucleotide identity and ≥90% coverage. A gene presence/absence matrix was generated for downstream epidemiological analyses. For reporting, virulence determinants were summarized as prevalence of the top 20–30 virulence genes and functional groupings (e.g., adhesion, iron acquisition, toxins, capsule/serum survival, invasion-related functions) based on database annotation and expert curation.

Isolates were classified as ExPEC-like using a predefined marker panel consistent with the “inclusive ExPEC” concept (e.g., ≥2 of *papA/papC*, *sfa/foc*, *afa/draBC*, *kpsM II*, *iutA*). DEC pathotypes were assigned using canonical marker logic (examples: STEC/EHEC—*stx1/stx2* ± *eae*; EPEC—*eae* with/without *bfpA*; ETEC—*elt/est*; EAEC—*aggR* and/or associated aggregative adherence markers; EIEC/Shigella—*ipaH/virF*). ExPEC-like status was assigned using the Russo and Johnson (18) marker panel, defined as the presence of ≥2 of the following markers: *papA/papC, sfa/foc, afa/dra, kpsM II*, *iutA*.

### 2.10 Phylogrouping and MLST

Phylogroups were inferred in silico based on the Clermont scheme using a genome-based implementation (e.g., EzClermont/ClermonTyping), and isolates were assigned to Clermont phylogroups (A, B1, B2, D, etc.) for comparative analyses. Multi-locus sequence typing (MLST) was performed using the Achtman 7-locus scheme (*adk, fumC, gyrB, icd, mdh, purA, recA*) and curated allele/ST databases (e.g., EnteroBase/PubMLST).

### 2.11 Statistical analysis and visualization

Analyses were conducted in R (v4.1) with standard statistical packages. Virulence gene load (per isolate) was defined as the number of detected virulence genes meeting the calling thresholds. Differences in virulence gene load across phylogroups and ESBL status were assessed using non-parametric tests (Kruskal–Wallis with *post hoc* Dunn testing) or generalized models as appropriate. Associations between categorical variables (e.g., ExPEC-like vs. non-ExPEC-like; DEC-like vs. non-DEC-like; hybrid vs. non-hybrid) and key AMR features (e.g., ESBL phenotype; presence of *mcr-1*; plasmid-borne fluoroquinolone resistance determinants such as *qnr* variants) were tested using Fisher’s exact test or χ^2^ tests with false discovery rate control (Benjamini–Hochberg). Correlations between plasmid and MGE-associated ARG burden and virulence signatures were quantified using Spearman’s rank correlation.

For visualization, phylogroup distributions and virulence burdens were presented as stacked bar plots and boxplots, respectively. Virulome heatmaps (isolates × virulence genes) were generated using hierarchical clustering (distance and linkage stated in figure legends), with annotation tracks for ESBL status and plasmid or MGE-associated ARG load. Co-occurrence patterns across virulence and AMR determinants were summarized using UpSet plots to highlight convergent and hybrid profiles.

All statistical tests were two-sided, and *p* < 0.05 was considered statistically significant unless stated otherwise. Where multiple comparisons were performed, *p*-values were adjusted using the Benjamini–Hochberg false discovery rate procedure.

## 3 Results

### 3.1 Virulome overview and virulence gene burden

**Figure 1:**
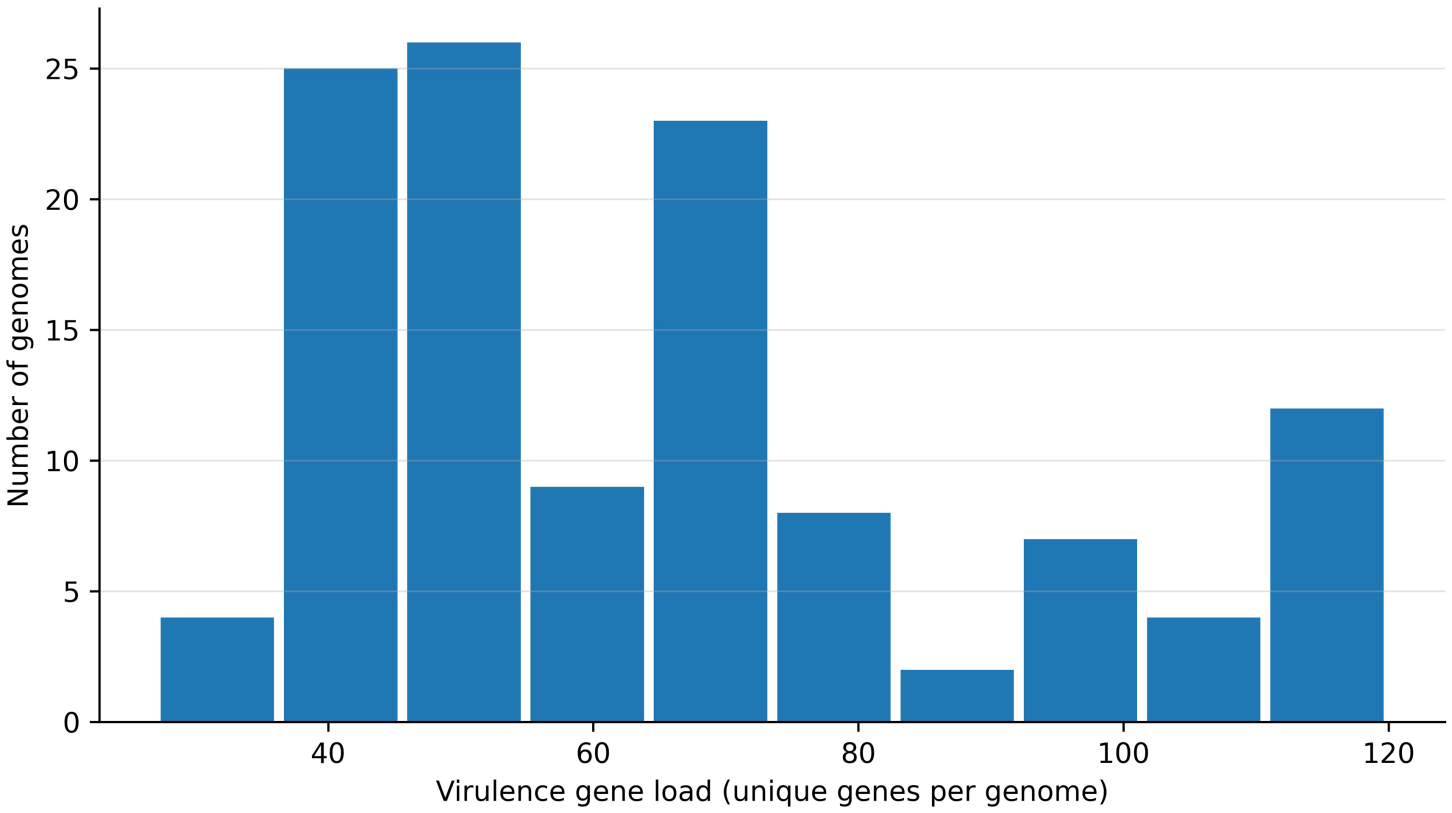
Distribution of virulence genes across swine-associated multidrug-resistant (MDR) *Escherichia coli* genomes. Virulence gene load was defined as the number of unique virulence-associated genes detected per genome in the virulence screening output (presence or absence at the gene level). The histogram illustrates the cohort-level heterogeneity in virulence gene content, highlighting a broad spread of virulence gene repertoires among isolates.

A total of 208 distinct virulence-associated genes were detected across the analytic WGS dataset (*n* = 116 genomes). At the isolate level, the virulence gene load (unique virulence genes per genome) was heterogeneous, with a median of 62 genes (IQR 46–78) and a range of 33–120. Only a small core set of determinants was ubiquitous across the cohort, whereas most genes showed variable presence, supporting marked strain-to-strain differences in virulence-associated genomic content (Figure 1).

**Figure 2:**
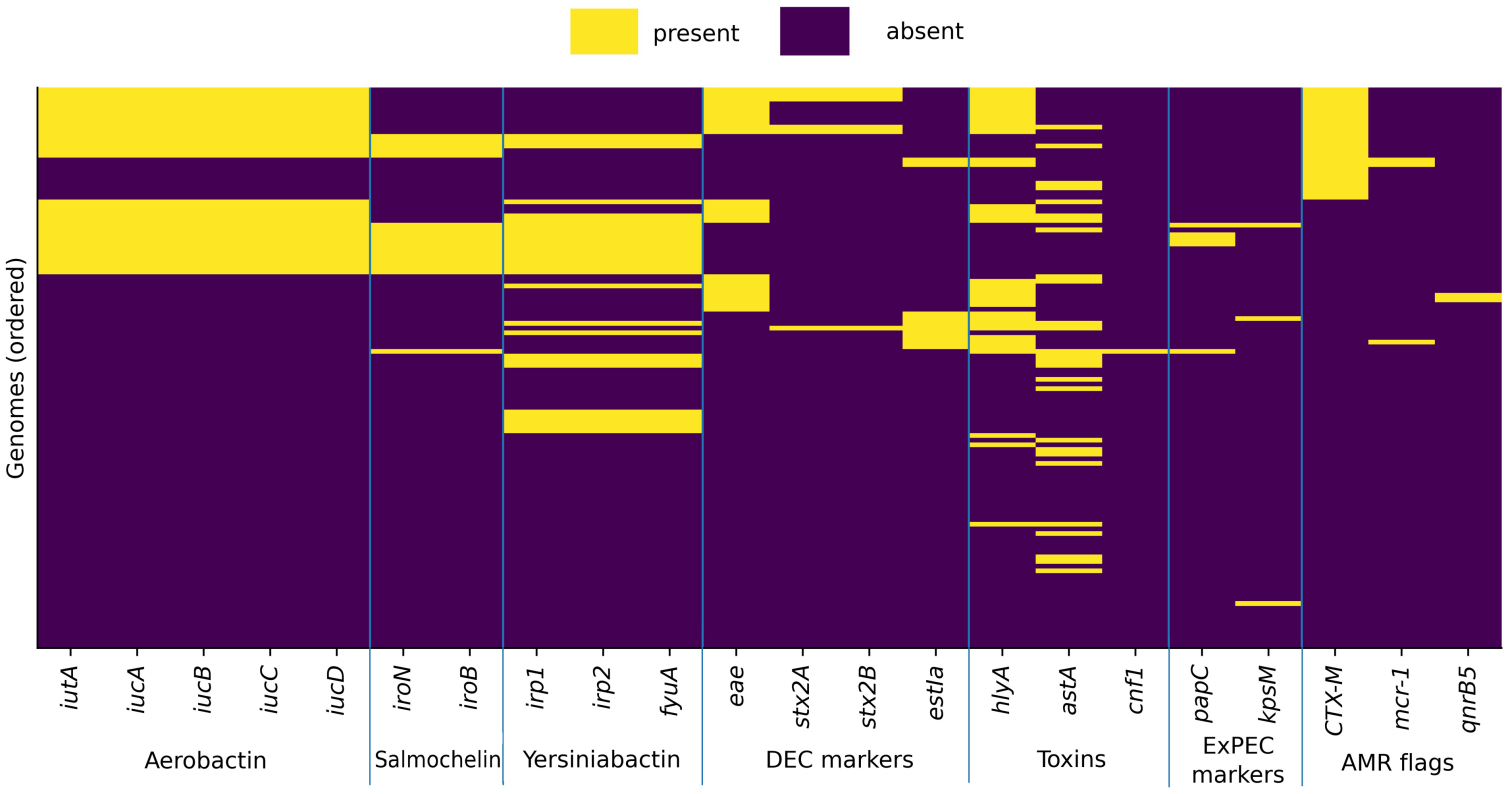
Heatmap of key virulence markers and selected high-impact resistance flags across swine-associated multidrug-resistant (MDR) *Escherichia coli* genomes. Columns indicate the presence or absence of selected virulence determinants representing major functional modules (iron acquisition: aerobactin *iucABCD/iutA*, salmochelin *iroB/iroN*, yersiniabactin *irp1/irp2/fyuA*; toxins/effectors: *hlyA*, *cnf1*, *astA*, *stx2A/stx2B*; intestinal attachment/toxins: *eae*, *estIa*; selected extraintestinal pathogenic *E. coli* (ExPEC)-associated markers: *papC*, *kpsM*), together with resistance “flags” limited to epidemiologically high-impact determinants (CTX-M family ESBL, *mcr-1*, and *qnrB5*). Rows represent individual genomes and were ordered to facilitate visual identification of convergent profiles (prioritized by *CTX-M* carriage, aerobactin module presence, diarrheagenic *Escherichia coli* (DEC) marker presence, and overall virulence gene load). This figure highlights patterns of co-carriage between virulence modules and selected resistance determinants without aiming to provide a comprehensive resistome analysis.

**Figure 3:**
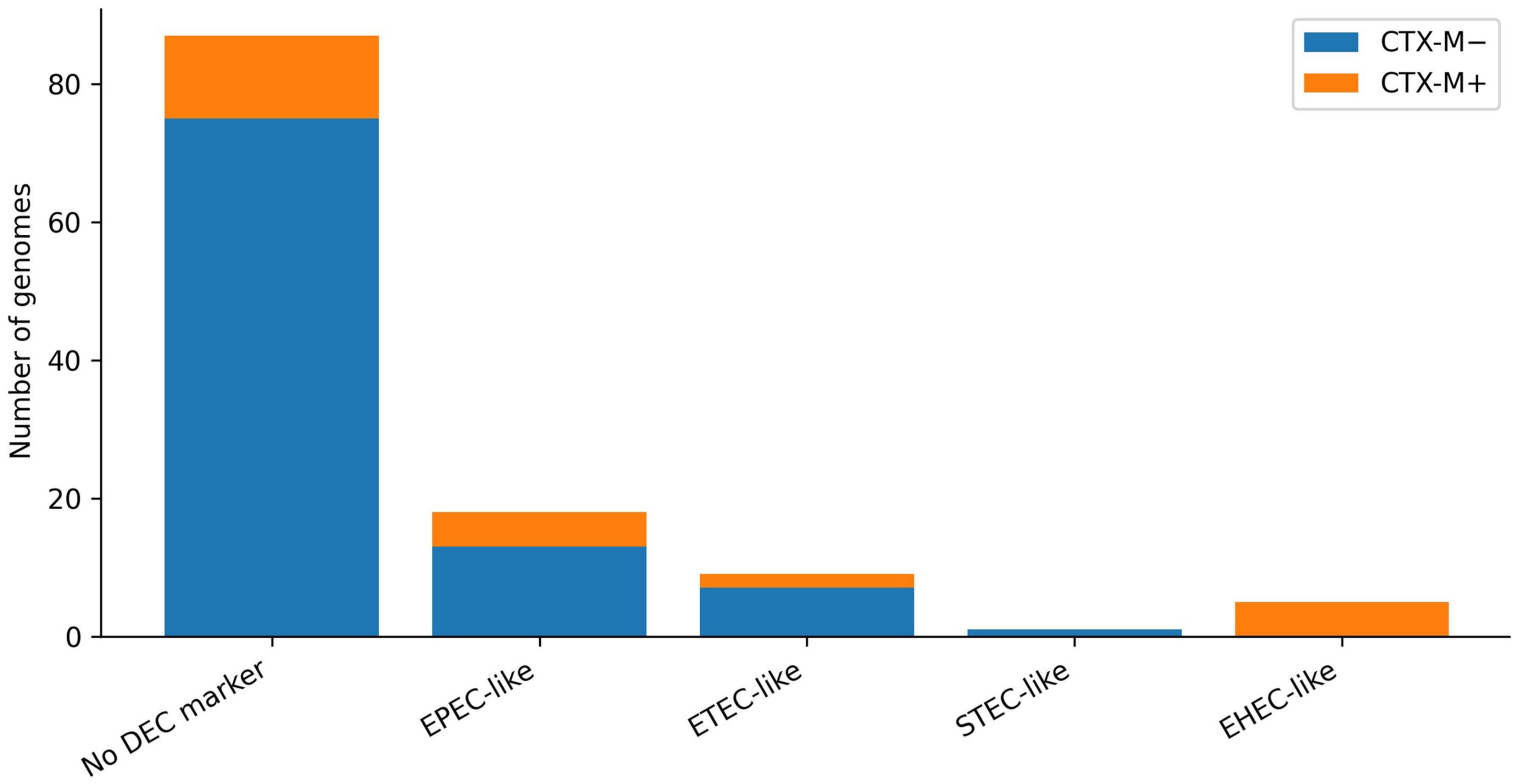
Marker-based diarrheagenic *Escherichia coli* (DEC) primary categories stratified by *CTX-M* carriage in swine-associated multidrug-resistant (MDR) *Escherichia coli* genomes. A hierarchical marker-based assignment was applied to summarize intestinal pathotype signals: enterohemorrhagic *Escherichia coli* (EHEC)-like (*stx2* and *eae*), shiga toxin-producing *Escherichia coli* (STEC)-like (*stx2* only), enteropathogenic *Escherichia coli* (EPEC)-like (*eae* only), enterotoxigenic *Escherichia coli* (ETEC)-like (*estIa*), and no DEC marker. Bars show the number of genomes within each category with (*CTX-M*+) or without (*CTX-M*−) *CTX-M* family ESBL genes. The plot provides a virulence-focused view of how key enteric marker patterns distribute across isolates with and without a major ESBL determinant.

Functionally, the virulome was dominated by determinants related to adhesion and biofilm formation and iron acquisition, with additional contributions from toxins and effector-like genes, consistent with a mixed population containing both commensal-adapted and potentially higher-risk virulence architectures. These patterns provided the basis for downstream marker-based interpretation of ExPEC- and DEC-associated signatures and for targeted integration with selected, high-impact resistance features. Functionally, adhesion/biofilm and iron acquisition determinants formed the dominant virulence backbone across the cohort, whereas toxin-associated and marker-defined pathotype signatures were restricted to distinct subsets. Table 1 summarizes the most prevalent virulence determinants and selected high-interest markers, while Table 2 consolidates marker-based ExPEC or DEC profiles together with the targeted high-impact resistance flags. Figures 1–3 provide an integrated visual summary of virulence gene load, marker co-carriage patterns, and DEC-category distribution stratified by *CTX-M* carriage.

**Table 1:** Most prevalent virulence-associated genes and selected high-interest markers detected in swine-associated multidrug resistant (MDR) *Escherichia coli* genomes.

Gene	Functional group	Representative annotation	Prevalence (*n* = 116)
*entA*	Iron acquisition	23-dihydro-23-dihydroxybenzoate dehydrogenase	116 (100.0%)
*entB*	Iron acquisition	Isochorismatase	116 (100.0%)
*entC*	Iron acquisition	Isochorismate synthase 1	116 (100.0%)
*entE*	Iron acquisition	23-dihydroxybenzoate-AMP ligase component of enterobactin synthase	115 (99.1%)
*entS*	Iron acquisition	Enterobactin exporter iron-regulated	115 (99.1%)
*fepA*	Iron acquisition	Ferrienterobactin outer membrane transporter	112 (96.6%)
*fepB*	Iron acquisition	Ferrienterobactin ABC transporter periplasmic binding	115 (99.1%)
*fepC*	Iron acquisition	Ferrienterobactin ABC transporter ATPase	116 (100.0%)
*fepD*	Iron acquisition	Ferrienterobactin ABC transporter permease	115 (99.1%)
*fes*	Iron acquisition	Enterobactin/ferric enterobactin esterase	114 (98.3%)
*fimC*	Adhesion/biofilm	Chaperone fimC precursor	107 (92.2%)
*fimF*	Adhesion/biofilm	FimF precursor	112 (96.6%)
*fimG*	Adhesion/biofilm	FimG precursor	113 (97.4%)
*fimH*	Adhesion/biofilm	FimH precursor	113 (97.4%)
*csgD*	Adhesion/biofilm	DNA-binding transcriptional regulator *CsgD*	112 (96.6%)
*csgG*	Adhesion/biofilm	Curli production assembly/transport *CsgG*	115 (99.1%)
*yagZ/ecpA*	Adhesion/biofilm	*E. coli* common pilus structural subunit *EcpA*	110 (94.8%)
*yagY/ecpB*	Adhesion/biofilm	*E. coli* common pilus chaperone *EcpB*	110 (94.8%)
*ompA*	Other	outer membrane A	116 (100.0%)
*espX4*	Toxins/effectors & secretion	Type III secretion system effector EspX4	115 (99.1%)
*espX5*	Toxins/effectors & secretion	Type III secretion system effector EspX5	116 (100.0%)
*nleH1*	Toxins/effectors & secretion	Type III secretion system effector NleH1	20 (17.2%)
*escN*	Toxins/effectors & secretion	ATPase EscN	23 (19.8%)
*tir*	Toxins/effectors & secretion	Translocated intimin receptor	12 (10.3%)
*iutA*	Iron acquisition	Ferric aerobactin receptor precursor IutA	31 (26.7%)
*iucA*	Iron acquisition	Aerobactin siderophore biosynthesis IucA	31 (26.7%)
*iucB*	Iron acquisition	Aerobactin siderophore biosynthesis IucB	31 (26.7%)
*iucC*	Iron acquisition	Aerobactin siderophore biosynthesis IucC	31 (26.7%)
*iucD*	Iron acquisition	L-lysine 6-monooxygenase IucD	31 (26.7%)
*iroB*	Iron acquisition	Glucosyltransferase IroB	17 (14.7%)
*iroN*	Iron acquisition	Salmochelin receptor IroN	17 (14.7%)
*irp1*	Iron acquisition	Yersiniabactin biosynthetic Irp1	27 (23.3%)
*irp2*	Iron acquisition	Yersiniabactin biosynthetic Irp2	27 (23.3%)
*fyuA*	Iron acquisition	Pesticin/yersiniabactin receptor	27 (23.3%)
*hlyA*	Toxins/effectors & secretion	Hemolysin toxin	33 (28.4%)
*eae*	Other	Intimin	23 (19.8%)
*stx2A*	Toxins/effectors & secretion	Shiga-like toxin II A subunit encoded by bacteriophage BP-933 W	6 (5.2%)
*stx2B*	Toxins/effectors & secretion	Shiga-like toxin II B subunit encoded by bacteriophage BP-933 W	6 (5.2%)
*estIa*	Toxins/effectors & secretion	Heat stable enterotoxin I	10 (8.6%)
*astA*	Toxins/effectors & secretion	Heat-stable enterotoxin 1	27 (23.3%)
*papC*	Adhesion/biofilm	Usher PapC	5 (4.3%)
*kpsM*	Capsule/surface protection	KpsM	3 (2.6%)
*cnf1*	Toxins/effectors & secretion	Cytotoxic necrotizing factor 1	1 (0.9%)

Genes are grouped by primary functional category and reported as counts and percentages of genomes positive for each determinant.

**Table 2:** Marker-based extraintestinal pathogenic/diarrheagenic *Escherichia coli* (ExPEC/DEC) profiles and selected high-impact resistance flags (CTX-M family ESBL, *mcr-1*, *qnrB5*) in swine-associated multidrug resistant (MDR) *E. coli* genomes.

Marker/profile (definition)	Isolates (*n* = 116)	*CTX-M* (n)	*mcr-1* (*n*)	*qnrB5* (*n*)
ExPEC-like (≥2-marker score: *papC/papA; sfa/foc; afa/dra; iutA; kpsM*)	6 (5.2%)	0	0	0
Aerobactin module (*iucABCD* + *iutA*)	31 (26.7%)	15	0	0
DEC marker-positive (*eae* and/or *stx2* and/or *estIa*)	33 (28.4%)	12	3	2
EPEC-like (*eae*+, *stx2*−)	18 (15.5%)	5	0	2
EHEC-like (*eae*+ and *stx2*+)	5 (4.3%)	5	0	0
STEC-like (*stx2*+, *eae*−)	1 (0.9%)	0	0	0
ETEC-like (*estIa*+)	10 (8.6%)	2	3	0
EAST1 toxin (*astA*+)	27 (23.3%)	4	0	0
α-hemolysin (*hlyA*+)	33 (28.4%)	12	3	2
Convergence subset (aerobactin + and DEC+)	15 (12.9%)	10	0	0

Profiles were assigned using predefined marker logic; values are reported as the number (and percentage) of genomes meeting each profile definition, with corresponding counts of selected resistance flags.

### 3.2 Iron acquisition systems and extraintestinal-associated virulence signals

Iron acquisition determinants were frequent, and several siderophore modules with established relevance to extraintestinal fitness and virulence were detected in a substantial subset of genomes. The aerobactin system (*iucABCD/iutA*) was detected in 31/116 genomes (26.7%), indicating that over one-quarter of isolates carried this ExPEC-associated iron uptake module. The salmochelin system (*iroB/iroC/iroD/iroN*) was observed in 17/116 (14.7%) and showed strong overlap with aerobactin: 16/17 salmochelin-positive isolates also carried aerobactin. The yersiniabactin cluster (*fyuA*, *irp1*, *irp2*) was detected in 27/116 (23.3%) and co-occurred with aerobactin in 17/31 aerobactin-positive isolates, suggesting recurrent accumulation of multiple iron acquisition systems in a subset of strains (Table 1).

Together, these data indicate a non-trivial fraction of swine-associated MDR *E. coli* carrying virulence-relevant siderophore repertoires that extend beyond baseline enterobactin-associated iron scavenging and may contribute to increased fitness and host interaction potential.

### 3.3 Adhesins and hemolysin-associated profiles

Adhesion and surface-associated determinants were widely represented, including gene sets consistent with broad intestinal colonization and biofilm-associated phenotypes. In addition, classical ExPEC-associated adhesin markers were present but comparatively rare. The P fimbrial structural marker *papC* was detected in 5/116 (4.3%), and capsule-associated *kpsM* in 3/116 (2.6%).

Notably, *hlyA* was detected in 33/116 genomes (28.4%), indicating a sizeable toxin-associated subset. A single genome carried *cnf1* (1/116; 0.9%), representing a rare but high-significance finding due to its established association with invasive extraintestinal disease phenotypes.

### 3.4 DEC-associated markers and intestinal pathotype signatures

Marker-based screening revealed that 33/116 isolates (28.4%) carried at least one canonical DEC-associated determinant (enteric toxin/adhesion signature), indicating that almost one-third of the cohort contained genomic hallmarks consistent with diarrheagenic *E. coli* pathotypes or related virulence modules (Figure 2).

The *eae* (intimin) was detected in 23/116 (19.8%), supporting EPEC/EHEC-like attachment-associated signatures. Shiga toxin genes consistent with *stx2* were detected in 6/116 genomes (5.2%). Among these, 5/6 isolates were *eae*-positive, yielding an EHEC-like (*stx2* and *eae*) profile in 5/116 (4.3%), while 1/6 represented an STEC-like (stx-only) profile. The ETEC-associated heat-stable toxin marker *estIa* was present in 10/116 (8.6%), supporting an ETEC-like signature subset. The EAST1 toxin gene *astA* was common (27/116; 23.3%), frequently co-occurring with other enteric virulence markers and contributing to an overall enrichment of toxin-associated virulence content in a subset of genomes (Figure 3).

These distributions demonstrate that swine-associated MDR *E. coli* in this cohort included multiple DEC-relevant virulence marker patterns, including a smaller but epidemiologically important *stx2*-positive subset.

### 3.5 ExPEC-like marker patterns and “convergent” virulence architectures

Using the predefined ExPEC marker logic (panel-based scoring), only a minority of isolates met stringent ExPEC-like criteria (6/116; 5.2%), reflecting that classical ExPEC marker constellations (e.g., P fimbriae, capsule combinations) were uncommon in this swine-associated MDR population. However, several isolates carried partial ExPEC-associated signatures, most prominently siderophore modules (aerobactin, salmochelin, yersiniabactin) and hemolysin, which are often interpreted as risk-elevating features when present in combination.

Importantly, 15/116 isolates (12.9%) co-carried aerobactin (ExPEC-associated iron acquisition) together with at least one DEC-associated marker (e.g., *eae*, *stx2*, and/or *estIa*). While these isolates did not necessarily satisfy strict ExPEC classification, this pattern represents virulence modularity and convergence, i.e., co-occurrence of extraintestinal-associated fitness/virulence systems with enteric pathotype markers, meriting targeted reporting and isolate-level interpretation.

### 3.6 ESBL-relevant *CTX-M* genes and virulence co-carriage

Among the 116 genomes, CTX-M family ESBL genes were detected in 24/116 isolates (20.7%). *CTX-M* carriage showed a strong, non-random association with aerobactin positivity: 15/31 (48.4%) of aerobactin-positive isolates carried *CTX-M*, compared with 9/85 (10.6%) of aerobactin-negative isolates (Fisher’s exact test OR = 7.92; *p* = 3.18 × 10^−5^). This finding supports a virulome–resistome convergence signal in which an ExPEC-associated siderophore module is enriched among ESBL-genotype carriers.

Similarly, isolates carrying any DEC marker were more likely to carry *CTX-M* (12/33 vs. 12/83; OR = 3.38; *p* = 0.0119). Strikingly, all EHEC-like (*stx2* and *eae*) isolates (5/5) carried *CTX-M*, highlighting a small but high-significance subset in which an important enteric virulence signature co-exists with ESBL-associated resistance.

### 3.7 Rare but critical determinants in virulence context

The plasmid-mediated colistin resistance determinant *mcr-1* was detected in 3/116 isolates (2.6%), and the plasmid-mediated quinolone resistance gene *qnrB5* in 2/116 (1.7%). Although low in prevalence, these isolates were not virulence-neutral: the *mcr-1* carriers in this dataset exhibited ETEC-like toxin signatures (*estIa*) and hemolysin co-carriage (*hlyA*), while *qnrB5* occurred in *eae*-positive isolates (EPEC-like). These observations support targeted follow-up of the genetic context of these determinants and reinforce the relevance of a virulence-informed interpretation of rare resistance flags in livestock-associated *E. coli* (Table 2).

## 4 Discussion

This study provides a virulence-focused genomic risk profile for swine-associated MDR or ESBL *E. coli* in Hungary by quantifying virulence gene burden, mapping ExPEC- and DEC-associated marker signatures, and testing whether these virulence architectures co-occur non-randomly with selected, high-impact resistance flags. Three findings stand out: the virulome was highly heterogeneous across genomes, consistent with a mixed population spanning primarily colonization-adapted lineages and a distinct subset enriched for higher-risk modules; extraintestinal-associated iron acquisition systems (particularly aerobactin, often with salmochelin and/or yersiniabactin) and toxin-associated signals (notably *hlyA*) were common enough to be epidemiologically meaningful; and virulence–resistance convergence was supported by a strong enrichment of *CTX-M* ESBL genes among aerobactin-positive isolates and among DEC-marker-positive isolates, including universal *CTX-M* co-carriage within the small EHEC-like (*stx2* and *eae*) subset. From a veterinary and One Health standpoint, these patterns are relevant for two reasons. First, the substantial prevalence of DEC markers in an MDR reservoir implies that enteric virulence modules circulate in production settings where antimicrobial exposure can indirectly stabilize linked mobile elements. Even when marker-based profiles are interpreted conservatively, the presence of *eae-, stx2*-, and/or *estIa*-associated signatures in a swine reservoir warrants risk-oriented interpretation because these modules can shape colonization dynamics, disease propensity in animal populations, and downstream exposure relevance across manure–environment and food-chain interfaces. Second, the enrichment of *CTX-M* among aerobactin-positive and DEC-marker-positive isolates suggests that clinically important *β*-lactam resistance is not distributed randomly but is concentrated in subsets carrying virulence-associated fitness modules, an architecture that can complicate mitigation because selection acting on one trait can help maintain the other within the same production ecosystem.

The overall distribution of virulence gene load (median 62 genes per genome, wide range) underscores that livestock-associated MDR *E. coli* cannot be treated as a uniform hazard class. The predominance of adhesion/biofilm and iron acquisition functions is biologically expected: these determinants support intestinal persistence, environmental survival, and competitive fitness under farm conditions. What is more informative is the uneven, modular accumulation of “risk-elevating” systems—iron-scavenging and toxin-associated features that are repeatedly implicated in host interaction and disease propensity in extraintestinal settings. In this sense, the cohort resembles a structured reservoir: most isolates carry a common colonization scaffold, while a non-trivial subset carries accessory modules that may materially shift risk under the right exposure pathways (occupational contact, manure–environment interfaces, and food-chain routes).

A key observation is the substantial prevalence of the aerobactin system (*iucABCD* and *iutA*) (26.7%), and its frequent co-occurrence with salmochelin (14.7%; mostly nested within aerobactin-positive genomes) and/or yersiniabactin (23.3%). Aerobactin and salmochelin are classically linked to virulence plasmids (often ColV, ColBM-like) and are repeatedly observed among ExPEC and related extraintestinal lineages, where enhanced iron acquisition can increase fitness in iron-limited host niches (58). The strong overlap between aerobactin and salmochelin in this dataset is consistent with the idea that these systems often travel together as a functional package rather than appearing independently, reinforcing the interpretation that a distinct, plasmid-linked virulence module is circulating in a subset of swine-associated MDR *E. coli*.

Yersiniabactin positivity (*fyuA, irp1, irp2*) adds a second layer of interpretation. The yersiniabactin “high-pathogenicity island” has long been associated with virulence-prone phylogenetic backgrounds (especially B2/D in human collections) and can contribute to both iron acquisition and broader stress/metal interactions (59). Although phylogroup/MLST stratification is the decisive step for attributing this signal to specific clonal backgrounds (and will matter for zoonotic inference), the present prevalence alone indicates that yersiniabactin-mediated fitness advantages are not rare in this production reservoir.

Classical ExPEC adhesin/capsule markers (e.g., *papC*, *kpsM*) were uncommon, and only 5.2% met stringent ExPEC-like criteria by the chosen panel logic. This “low ExPEC-score” outcome should not be over-interpreted as low virulence potential. Marker-panel definitions are intentionally conservative and may miss alternative, lineage-specific virulence configurations. At the same time, the frequent detection of *hlyA* (28.4%), usually accompanied by the broader operon, identifies a sizeable toxin-associated subgroup. Hemolysin is a well-established contributor to tissue damage and inflammatory pathology in extraintestinal contexts, and its presence—particularly when paired with enhanced iron acquisition—can be risk-elevating even when canonical ExPEC adhesin/capsule combinations are absent. The single detection of *cnf1* (0.9%) is rare but important, because *cnf1* is typically enriched among invasive extraintestinal lineages and often signals a “high consequence” virulence background even at low prevalence.

Nearly one-third of genomes carried at least one canonical DEC marker, with *eae* present in ~20%, *stx2* in ~5% (mostly with *eae*), and *estIa* in ~9%. These frequencies point to a heterogeneous enteric-virulence landscape within an MDR reservoir. For pigs specifically, interpretation depends on the detailed toxin/adhesion context and subtyping. Shiga-toxin-associated disease in swine is most classically linked to *stx2e*-mediated edema disease, while *eae* signals LEE-encoded attaching/effacing potential (EPEC/EHEC-like signatures). Recent experimental and field literature continues to emphasize that “hybrid” STEC/ETEC configurations can occur in pigs and can be clinically consequential in post-weaning disease ecology (60).

The comparatively frequent detection of *astA* (EAST1) also merits careful interpretation. EAST1 is widely distributed in porcine *E. coli* populations and is often considered an accessory toxin rather than a decisive pathotype hallmark; it can co-occur with fimbrial and enterotoxin profiles and may contribute to disease severity in some contexts, but it is not, by itself, a specific signature of EAEC (61, 62). In this cohort, *astA* appears to contribute to an overall toxin-enriched subset and may serve best as a “supporting” signal, most informative when interpreted alongside primary DEC markers (e.g., *eae*, *stx2*, *estIa*) and, where available, fimbrial adhesin typing and clinical metadata.

The strongest “convergence” signal in the results is the non-random association between *CTX-M* ESBL genes and virulence modules: CTX-M was markedly enriched among aerobactin-positive isolates (OR = 7.9) and was also more frequent among DEC-marker-positive isolates (OR = 3.4). The universal CTX-M co-carriage in the EHEC-like subset (5/5) is especially noteworthy, because it unites two traits of high public-health concern: an attachment/toxin signature classically associated with severe enteric disease potential, and an ESBL genotype that compromises critically important *β*-lactams.

Mechanistically, the observed enrichment of CTX-M among isolates carrying aerobactin-centered modules and DEC markers is consistent with plasmid-centric co-carriage and co-selection. Resistance and virulence determinants may be maintained on shared mobile backbones or co-stabilized within the same strain under antimicrobial pressure in production environments. While short-read data do not allow definitive inference of physical linkage, the convergence signal supports targeted follow-up using plasmid reconstruction (long-read sequencing) and local genomic context analysis around *CTX-M* and siderophore loci.

In European pig systems, the co-occurrence of ESBL genotypes with mixed virulence signatures has been reported, including combinations of ExPEC-associated factors and enterotoxin-associated genes that suggest hybrid or modular virulence architectures in swine-associated ESBL *E. coli* (34). Our dataset extends this concept by quantifying the magnitude of aerobactin-centered modules in a larger cohort and by statistically demonstrating enrichment of *CTX-M* within these virulence-positive subsets.

The detection of *mcr-1* (2.6%) and *qnrB5* (1.7%)—even at low prevalence—has disproportionate One Health relevance because these genes can spread horizontally and erode therapeutic options (colistin as a last-resort agent; fluoroquinolones as critically important drugs). Reports from diverse settings continue to document *mcr*-carrying ESBL *E. coli* spanning animal and human sources, emphasizing that livestock-associated reservoirs can interface with human risk pathways (26). The present observation that these determinants occur in isolates that are not virulence-neutral (ETEC-like plus hemolysin in *mcr-1* carriers; *eae* positivity in *qnrB5* carriers) further supports a risk-based surveillance stance: rare resistance flags should be interpreted in the context of virulence modules and potential exposure routes, not as isolated genetic events.

From a One Health surveillance perspective, the key implication is not merely that “virulence genes exist” in swine-associated MDR *E. coli*, but that virulence-relevant modules (iron acquisition, toxins, DEC markers) appear in specific combinations and show measurable co-occurrence with ESBL genotypes. This reinforces the rationale for integrated monitoring frameworks that track both resistance and virulence in indicator *E. coli* from food animals, as also reflected in EU-level AMR surveillance emphasis on commensal *E. coli* and ESBL/*ampC* monitoring in pigs (36).

This observational cross-sectional study is isolate-based and restricted to culture-positive submissions generated through routine diagnostic/surveillance activities; accordingly, it does not estimate farm-level prevalence and cannot capture culture-negative submissions. The dataset originates from four large-scale farms within a defined period, and animal-level clinical metadata were not uniformly available, limiting inference on clinical correlations of virulence-marker carriage. Whole-genome sequencing was performed on a subset of the phenotyped collection, constrained by DNA quality and genome QC, which may introduce selection effects relative to the full cohort. Virulence inference is gene-centric and does not measure expression, regulation, or phenotypic consequences, and short-read data preclude definitive reconstruction of plasmid architectures and physical linkage between *CTX-M* and virulence modules. Finally, toxin subtyping and higher-resolution serotype/plasmid resolution were beyond the present scope and should be prioritized in follow-up work to strengthen risk attribution.

## 5 Conclusion

Taken together, these data show that swine-associated MDR *E. coli* from Hungary exhibit pronounced virulome heterogeneity, with frequent carriage of aerobactin-centered iron acquisition (26.7%) and toxin-associated signals (including *hlyA* in 28.4%), alongside substantial DEC-marker prevalence (28.4%) and a small *stx2 + eae* (EHEC-like marker) subset (4.3%). The enrichment of *CTX-M* ESBL genes among aerobactin-positive and DEC-marker-positive isolates supports virulome–resistome convergence within a production reservoir and reinforces the value of integrated One Health surveillance that interprets resistance flags in the context of virulence modules and marker-defined risk backgrounds. These findings motivate targeted follow-up using plasmid resolution and refined toxin or serotype characterization to strengthen risk attribution.

## Data Availability

The datasets presented in this study can be found in online repositories. The names of the repository/repositories and accession number(s) can be found in the article/Supplementary material.
