# Supplementary figures and images for: Virulome–resistome convergence in swine-associated multidrug-resistant Escherichia coli from Hungary: virulence marker profiles and zoonotic potential
Source: Front Vet Sci. 2026 Apr 16;13:1813532. doi: 10.3389/fvets.2026.1813532 (PMC13128422; doi:10.3389/fvets.2026.1813532)

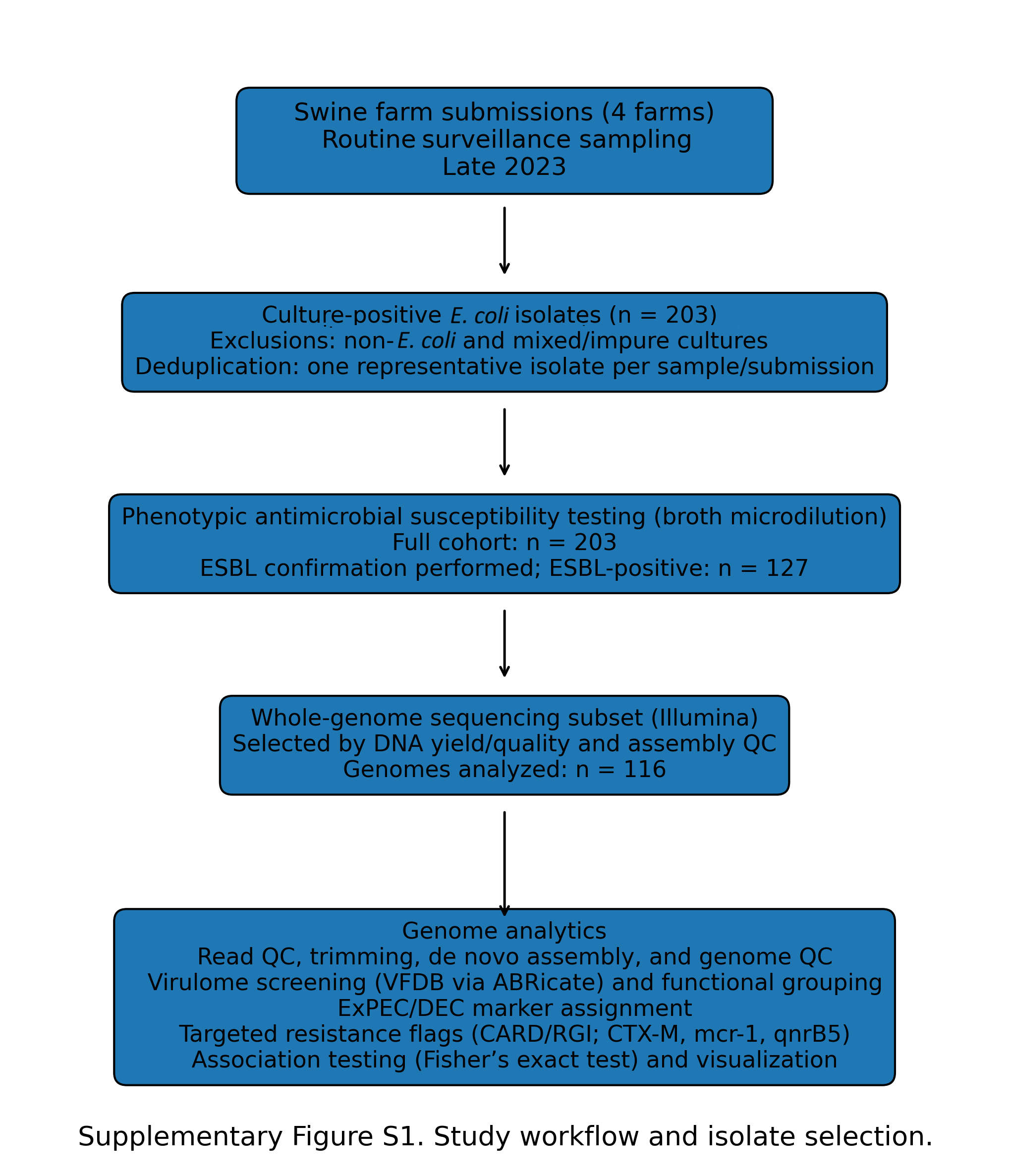

Supplement: Supplementary file 1 [file Image_1.PNG]
